# The relationship between principal instructional leadership and teacher self-efficacy in student engagement and classroom management: a cross-sectional study in China

**DOI:** 10.3389/fpsyg.2025.1589958

**Published:** 2025-08-20

**Authors:** Naiyuan Zhang, Yan-Li Siaw, Na Jiang

**Affiliations:** ^1^School of Education, South China Normal University, Guangzhou, China; ^2^Faculty of Education, University of Malaya, Kuala Lumpur, Malaysia; ^3^Faculty of Teacher Education, Shangrao Normal University, Shangrao, China

**Keywords:** teacher self-efficacy, student engagement, principal instructional leadership, student management, cross-sectional study

## Abstract

**Introduction:**

This study examines the relationship between principal instructional leadership and teacher self-efficacy in student engagement and classroom management within Chinese primary and secondary schools. Grounded in Bandura’s self-efficacy theory, it addresses two research questions: (1) What is the association between principal instructional leadership and teacher self-efficacy? (2) Which specific leadership dimensions most significantly predict teacher efficacy?

**Methods:**

A quantitative research design was employed, with data collected from 459 teachers through two validated online instruments: the Educational Leadership Instrument (ELI) and the Teachers’ Sense of Efficacy Scale (TSES). Data analysis included descriptive statistics, correlation analysis, and multiple regression.

**Results:**

Results demonstrated significant positive correlations between instructional leadership and teacher self-efficacy (*r* = 0.75–0.84, *p* < 0.01). Regression analysis identified “work environment support” (*β* = 0.488) and “teacher-student engagement promotion” (*β* = 0.518) as the strongest predictive dimensions of teacher efficacy.

**Discussion:**

The findings underscore the pivotal role of instructional leadership in enhancing teachers’ confidence in student engagement and classroom management. This study contributes to leadership literature by highlighting culturally relevant dimensions in non-Western educational contexts, while offering practical implications for principal training programs and professional development initiatives.

## Introduction

Over recent decades, the roles of school principals have evolved significantly, influenced by changes in educational philosophies, increasing societal expectations, greater complexity in school management, and the impact of educational policies (Hallinger and Walker, 2017; Daniëls et al., 2019; Redondo-Sama et al., 2025). Hence, the role of principals has shifted from being administrative managers to being instructional leaders and agents of change. Across the globe, instructional leadership has become a central strategy for improving teaching quality, fostering positive teaching culture, supporting teachers’ professional development, and enhancing students’ academic performance (Hallinger, 2008; Hallinger and Heck, 2010; Liu et al., 2022; Berkovich and Hassan, 2024).

In parallel, a number of studies highlighted that teacher self-efficacy is crucial in determining teaching practices, enriching students’ learning experiences, and improving learning outcomes (Berman et al., 1977; Guskey and Passaro, 1994; Hettinger et al., 2024; Sellami et al., 2025). Teachers with higher self-efficacy often demonstrated greater teaching motivation, innovation, and perseverance, which contributed significantly to improved teaching quality and student achievement (Guskey, 1984; Allinder, 1994; Kasalak and Dagyar, 2020; Röhl et al., 2024). Furthermore, previous studies have also emphasised the pivotal role of school leadership behaviour and style in shaping teacher self-efficacy. Given these dual developments, understanding how principal instructional leadership influences teacher self-efficacy has become a critical issue for educational research and practice.

However, despite the increasing number of studies focusing on principal leadership and teacher self-efficacy, most of the studies on teacher self-efficacy have been conducted in Anglo-American knowledge systems (Mertkan et al., 2017; Hallinger and Kovačević, 2019; Ma and Marion, 2021). Therefore, studies examining the relationship between principal leadership and teacher self-efficacy within the Chinese educational context remains limited. Furthermore, within China’s unique cultural framework, the educational system and traditions have a more intricate impact on teacher self-efficacy. Chinese school leaders often occupy conventional authoritative roles, which significantly influence teachers’ professional confidence and classroom management strategies (Wong, 2003; Liu and Hallinger, 2021).

Addressing these gaps, this study examines the relationship between principal instructional leadership and teacher self-efficacy in student engagement and classroom management, using empirical data from primary and secondary schools in Jiangxi Province, China. Adopting a cross-sectional design, this study examined the direct and indirect effects of principal leadership practices on teacher self-efficacy. However, due to its cross-sectional nature, the study cannot establish causality between the variables. Future research should consider longitudinal or experimental designs to validate these relationships over time. The empirical data analysis provided an in-depth perspective on the dynamic interplay between leadership and self-efficacy within China’s unique cultural and institutional framework. The findings contributed to the enhancement of teaching quality, providing innovative insights into effective educational leadership practices, and supporting educational reform in improving teaching effectiveness and student engagement. Furthermore, this study also provided a more comprehensive perspective on teacher behaviour and the cultural nuances of educational environments.

Additionally, this study contributes to the literature in three key ways. First, it focuses on two critical dimensions of teacher self-efficacy: student engagement and classroom management which have not been sufficiently differentiated in prior Chinese studies. Second, it applies Bandura’s theoretical framework in the Chinese educational context, offering insights into how cultural values such as hierarchical authority and collectivism influence the leadership-efficacy link. Third, it provides robust quantitative evidence based on a large sample of Chinese primary and secondary school teachers, addressing the existing gap in non-Western empirical research on instructional leadership.

Despite the growing interest in leadership and teacher efficacy, limited empirical research has investigated how instructional principal leadership influences teacher self-efficacy in China’s cultural and institutional context (Ma and Marion, 2021; Ahn and Bowers, 2024). Most studies in the Chinese context have remained descriptive, lacking robust quantitative models. Therefore, this study addresses the following hypotheses: (1) there is a significant positive correlation between principals’ instructional leadership and teacher self-efficacy; (2) principals instructional leadership significantly predicts teacher self-efficacy in student engagement and classroom management.

### Self-efficacy and teacher effectiveness

Self-efficacy is defined as an individual’s set of beliefs that determine how one can successfully execute a plan of action in prospective situations (Bandura, 1977). Self-efficacy influences behaviour choices, performance quality, effort levels, perseverance, and emotional and cognitive responses to challenges (Bandura, 1977). Furthermore, Bandura emphasised that self-efficacy is shaped by personal, social, emotional, and psychological factors. Zee and Koomen (2016) further argued that self-efficacy determines the goal setting and the actions taken to achieve them. Self-efficacy is not independent but is significantly affected by external environmental factors.

Bandura’s Self-Efficacy Theory identified four dimensions that are crucial in shaping self-efficacy. The first and most influential dimension is mastery experiences, which refers to the experiences that enhance confidence. The second dimension, vicarious experiences, involve observing other people’s performance and outcomes to develop self-assessments and confidence. Verbal persuasion refers to external encouragement or feedback that reinforces beliefs in task success. Finally, physiological and emotional states, such as stress, anxiety, confidence, or excitement, shape perceptions of capability through physical and emotional responses to tasks. These four dimensions interact dynamically and collectively shape efficacy beliefs. These dimensions are essential for understanding and strengthening teacher efficacy, providing theoretical foundation to enhance teachers’ confidence and effectiveness.

Teacher self-efficacy refers to teachers’ beliefs in their ability to impact students’ learning behaviours and academic outcomes throughout the teaching process. Tschannen-Moran et al. (1998, p. 233) described teacher self-efficacy as “a teacher’s belief in his or her own ability to organise and execute courses of action essential to successfully achieving specific teaching tasks in specific situations.” This includes the belief in and capacity to plan, organise, and implement necessary actions to accomplish instructional goals (Bandura, 1977; Donohoo, 2018).

Teachers with high self-efficacy exhibit greater confidence and resilience in overcoming instructional challenges. They employ effective teaching strategies and classroom management skills, demonstrating adaptability to various teaching scenarios (Lazarides et al., 2020; Ahn and Bowers, 2024). Furthermore, these teachers foster a positive classroom environment which enhances interactions with students. Studies indicated that teacher self-efficacy significantly influences teaching quality, school climate, and student outcomes (Soodak and Podell, 1993). Teachers with high self-efficacy are better equipped to manage challenging student behaviours, demonstrate higher empathy and persistence, and embrace innovative instructional methods (Klassen and Chiu, 2011; Mok and Moore, 2019). They also display greater overall effectiveness in their teaching practices (Klassen and Tze, 2014; Wang and Pan, 2023).

Teacher self-efficacy comprises two dimensions: (1) instructional efficacy, which involves the ability to inspire and motivate students to learn, and overcoming external factors such as socioeconomic background; and (2) personal efficacy, which relates to teachers’ beliefs in their capacity to implement critical instructional behaviours that impact student learning (Ashton and Webb, 1986). Empirical studies (e.g., Bandura, 1997; Mok and Moore, 2019) highlighted the significant impact of teacher self-efficacy on instructional practices, classroom management, and student engagement. Teachers with high self-efficacy are more likely to encourage student participation, enhancing learning motivation and outcomes (Wang and Pan, 2023). Conversely, teachers with low self-efficacy may exhibit anxiety and unease, leading to low classroom engagement (Zee and Koomen, 2016; Chen et al., 2024).

Effective classroom management is another essential aspect of successful teaching. Teachers with high self-efficacy demonstrated greater confidence in maintaining order, establishing positive teacher-student relationships, and promoting peer collaboration. These teachers are better equipped to handle unforeseen situations, minimise disruptions and ensure smoother instructional activities (Bandura, 1977; Shah, 2023; Duan et al., 2024). Hence, teacher self-efficacy is vital for teaching effectiveness and a key driver of educational improvements in creating a conducive learning environment and systemic progress.

### Principal leadership on teacher self-efficacy

The role of principal leadership in enhancing teaching quality, promoting teacher professional growth, and shaping school culture has been widely recognised by many researchers (Bandura, 1993; Blase and Blase, 1999; Hallinger, 2008). Principal leadership aims to enhance educational outcomes by designing and implementing student-centred teaching strategies. It focuses on the principal’s ability to establish relevant instructional goals tailored to the specific needs of the school and guide all staff towards achieving shared objectives (Leithwood and Sun, 2018). This perspective highlighted the importance of instructional leaders in creating supportive environments for educational development and student success.

Previous studies emphasised that principal leadership involves several key elements and behaviours. Hallinger (2011) examined the role of principals in implementing student-centred teaching strategies and highlighted the importance of their leadership in creating a conducive environment for educational growth and student success. He also emphasised the importance of establishing educational visions and concrete goals to guide teachers and students towards shared aspirations. Akram et al. (2017) and Murphy (1988) highlighted that principals play a vital role in providing essential resources and support to teachers and students to promote learning and development. Other studies highlighted the need for principals to continually refine teaching methods and curriculum content through regular evaluation and feedback (Sebastian and Allensworth, 2012; Husain et al., 2021). By integrating these elements, principals can effectively guide teachers and students towards optimal teaching and learning practices (Murphy et al., 2007; Moss and Brookhart, 2019).

Studies indicated that principal leadership can either strengthen or undermine teachers’ sense of efficacy. When principals effectively fulfilled their instructional leadership roles—such as creating favourable teaching conditions, providing clear instructional guidance, ensuring adequate resources, and offering positive feedback—they enhanced teachers’ self-esteem, motivation sense of efficacy, and instructional practices (Supovitz et al., 2010; Tschannen-Moran and Gareis, 2015). Conversely, ineffective instructional leadership—such as unclear goals, inadequate resource allocation, or lack of meaningful feedback—lead teachers to doubt their teaching abilities and reduce their sense of efficacy (Hallinger and Heck, 1996; Darling-Hammond et al., 2017). For example, frequent changes in instructional requirements without sufficient training and support can leave teachers feeling lost and powerless, ultimately undermining their confidence in effective teaching.

### Multidimensional nature of principal leadership

Principal leadership plays a crucial role in modern school management. Studies by Heck et al. (1990) and Sergiovanni (1984) suggested that instructional leadership is inherently multidimensional. These dimensions can be explored through leadership vision, teaching and learning management, environmental and resource management, and teacher engagement. Collectively, these dimensions form the comprehensive roles of principals in enhancing teaching quality and supporting teacher development. Based on the literature and theoretical frameworks, we hypothesised a positive correlation between the four aspects of instructional leadership—leadership vision, teaching and learning management, environmental and resource management, and teacher engagement—and teacher efficacy.

### Leadership vision

Leadership vision is the cornerstone of principal leadership, determining the strategic direction and values in school development. Sergiovanni (1990) and Leithwood et al. (2008) argued that principals need to establish clear school missions and goals to guide the collaborative efforts of teachers and students. For example, principals can ensure alignment between instructional activities and the school’s objectives by setting goals such as “student-centred learning” or “enhancing teacher professional development.” Leithwood and Sun (2018) emphasised that an effective leadership vision can subtly influence school culture through daily decisions and actions, promoting shared educational beliefs and goals among all members. A progressive leadership vision can drive innovation in teaching practices, motivating teachers to explore new methods and embrace change.

### Teaching and learning management

Managing teaching and learning is a core responsibility of principal leadership. Effective instructional leadership enhances teachers’ professional development, strengthens teaching capabilities, and addresses learning challenges. Hallinger and Heck (1996) emphasised that principals must establish educational goals and lead and support teachers to ensure their effective implementation. This involved regularly observing classroom teaching, providing feedback and suggestions to help teachers improve instructional strategies (Balyer and Özcan, 2020). Wayman and Stringfield (2006) demonstrated that principals can identify weak points in teaching and implement targeted measures by analysing data on students’ performance and classroom engagement. Additionally, Hallinger and Heck (1996) emphasised the principal’s role in curriculum design and resource support, ensuring the curriculum aligns with students’ needs while providing necessary instructional resources, such as organising school-based research activities and introducing educational technology.

### Environmental and resource management

Principals influence teaching processes and outcomes by fostering supportive educational environments and optimising resource allocations (Spillane et al., 2001; Hallinger and Heck, 1996). According to Wang and Degol (2016), a safe, orderly, and motivating school environment enhances teacher job satisfaction and improve student learning outcomes. To support these goals, principals must effectively allocate resources—human, financial, and material—by prioritising initiatives like teacher training and updating teaching equipment to address instructional needs. Principals who foster a culture of trust and collaboration within the school significantly enhanced teachers’ sense of belonging and responsibility which increased their instructional commitment (Leithwood and Jantzi, 2005; Morris et al., 2020).

### Teacher engagement

Teacher engagement is considered a fundamental element in achieving school educational goals and a key aspect of principal leadership. By involving teachers in school decision-making, principals enhanced their sense of ownership and improve their enthusiasm and work motivation (Hallinger and Heck, 1996; Leithwood and Sun, 2018). Tschannen-Moran and Gareis (2015) further pointed out that such participation extends beyond routine teaching activities, encompassing broader domains like curriculum reform and school development planning. Principals should provide professional training and career development opportunities to help teachers consistently enhance their teaching skills and professional competencies to strengthen their self-efficacy and confidence. Moreover, principals can foster collaboration and shared purpose among teachers by organising team-based and interdisciplinary research activities that can enhance engagement and professional identity, such as teacher communities or instructional collaboration teams (Blase and Blase, 1999; Hallinger and Heck, 2010). In such environments, teachers can collaborate to refine teaching methods, share pedagogical experiences, and enhance teaching quality, ultimately achieving the school’s educational objectives.

### Objectives of the study

This study aimed to examine the influence of principal leadership on teacher self-efficacy in student engagement and classroom management. The two main objectives of the study are: (1) to investigate the correlation between principal leadership (leadership vision, teaching and learning management, environmental and resource management, teacher engagement) and teacher self-efficacy (efficacy in student engagement and classroom management), and (2) to examine the influence of principal leadership (leadership vision, teaching and learning management, environmental and resource management, teacher engagement) on teacher self-efficacy.

## Methodology

### Research design and data analysis

The purpose of the study was to examine the relationship and impact of principal leadership on teacher self-efficacy in China’s primary and secondary schools. In this quantitative study, two sets of online survey instruments were utilised to collect the information from a total of 459 teachers. Participants represented both urban and rural areas and varied in years of teaching experience and grade levels taught. To enhance the representativeness of the sample, a random sampling method was employed to select in-service teachers from schools across Jiangxi Province. Data collection was conducted between March and April 2024. Data were collected through an anonymous online survey distributed via a secure digital platform (*Wenjuanxing*). The survey link was shared with school leaders, who voluntarily disseminated it to their teaching staff. The purpose of the study was indicated on the front page of the survey and the respondents were assured that the survey collected no identifying information. All participation were voluntary and there were no incentives offered to the respondents. To minimise missing data and ensure quality responses, the survey platform required completion of all items before submission. On average, respondents took 10–12 min to complete the questionnaire.

The collected data were then analysed using descriptive and inferential statistical methods. The descriptive analysis provided a summary of the data, while correlation and multi-regression analysis were conducted to examine the relationship between the variables and the domains, and determined the predictors and assessed the overall contribution of independent variables to the dependent variables. Additionally, variance inflation factor (VIF) scores were examined for multicollinearity, and all predictors reported acceptable VIF values, indicating no issues of multicollinearity and suggesting the model assumptions were met.

### Respondents

The respondents of this study were 459 teachers from Jiangxi, China. This study selected Jiangxi as the research site because, as a central Chinese province, it represents the educational characteristics of moderately developed regions with urban–rural disparities and policy relevance, while ensuring data accessibility. If conducted in developed areas (e.g., Beijing), principals might emphasise innovative teaching with stronger resource support for teacher efficacy, whereas in underdeveloped western regions (e.g., Gansu), leadership impact could be constrained by systemic challenges. However, further multi-regional studies are recommended to enhance generalizability. The majority of the respondents were female (*n* = 326; 71%) and nearly half (*n* = 208; 45%) were aged 30 and below. In terms of educational level, most respondents held a Diploma/Foundation in Education (*n* = 313; 68.2%). Nevertheless, only 34.8% (*n* = 160) had more than 15 years of teaching experience. Among the respondents, 59.75% (*n* = 274) were national primary school teachers, and 40.3% (*n* = 185) were national secondary school teachers. Table 1 presents the detailed summary of the respondents’ demographic information.

**Table 1:** Demographic information.

Item	Frequency (%)
Gender	
Male	133 (29.0%)
Female	326 (71.0%)
Education level	
Diploma/Foundation	313 (68.2%)
Bachelor	132 (28.8%)
Master	10 (2.2%)
PhD	2 (0.4%)
Others	2 (0.4%)
Age	
30 and below	208 (45.3%)
31–40 years old	114 (24.8%)
41–50 years old	95 (20.7%)
51–60 years old	41 (8.9%)
61 and above	1 (0.2%)
Teaching experience	
5 years and below	189 (41.2%)
6–15 years	110 (24.0%)
16–25 years	85 (18.5%)
25 years and above	75 (16.3)

*N* = 459.

### Instrumentation

Two sets of online survey instruments were used to measure principal leadership and teacher self-efficacy: the Educational Leadership Instrument and Teacher’s Sense of Efficacy Scale.

#### Educational leadership

The Educational Leadership Instrument (ELI) (Siaw et al., 2021) was used to measure principal leadership across four dimensions, encompassing eight aspects as outlined in Table 2. The instrument consisted of 70 items and employed a five-point Likert scale. The reliability coefficients reported ranged from 0.96 to 0.98, indicating a high level of reliability (Fimian and Fastenau, 1990). The CFA results reported composite reliability (CR) values ranging from 0.96 to 0.97 and average variance extracted (AVE) values between 0.70 and 0.78, indicating excellent construct validity. Model fit indices (e.g., RMSEA = 0.083; CFI = 0.817; IFI = 0.817) confirmed the instrument’s suitability in measuring principal instructional leadership in Chinese schools.

**Table 2:** Educational leadership instrument—dimensions, aspects and reliability.

Dimension	Aspect	Item(s)	Cronbach’s Alpha
Leadership mindset	Mission goal	7	0.960
	Behaviour and personality	9	0.974
Teaching and learning	Curriculum and teaching	8	0.979
	Supervision and evaluation	10	0.984
Work environment	Learning and teaching	10	0.982
	Resources plan	8	0.972
Teacher and student	Teacher-centred	8	0.973
	Student-centred	10	0.982

#### Teacher self-efficacy

Teacher Self-Efficacy was assessed using Teachers’ Sense of Efficacy Scale (Tschannen-Moran and Hoy, 2001). Teacher’ Sense of Efficacy Scale is an established instrument which Duffin et al. (2012) have examined the CFA and showed good fit, high inter-factor correlations. The instrument consisted of 21 items across two dimensions: efficacy in student engagement and efficacy in classroom management. Responses were recorded on a five-point Likert scale and the reliability coefficients, as reported in Table 3, were deemed acceptable (Fimian and Fastenau, 1990).

**Table 3:** Teacher self-efficacy instrument—dimensions, aspects and reliability.

Dimension	Item(s)	Cronbach’s Alpha
Efficacy in student engagement	11	0.979
Efficacy in classroom management	10	0.976

Prior to conducting regression analysis, all survey data were screened for missing values, outliers, and normality. The assumptions of linearity, multicollinearity, and homoscedasticity were checked and met. The normality test was performed for all the variables. Results showed that the data collected was normally distributed, with the skewness ranged from 0.19 to 0.46 while the kurtosis values ware ranged from 0.16 to 0.55 (Witte and Witte, 2017). While, the VIF values are reported within 1.04 to 3.12, which are less than 5, indicating the collinearity are acceptable (Kiernan, 2014). This study opted for multiple linear regression due to its clarity in estimating the direct effect of each leadership dimension on specific self-efficacy outcomes, which aligns with the exploratory nature of this study.

## Results

### Principal leadership and teacher self-efficacy

The findings of the study indicated that majority of the respondents reported a high level of self-efficacy (*n* = 418; 91.1%) based in Pallant (2016) levels’ explanation. Additionally, most of the respondents stated that their principals demonstrated strong leadership, effective teaching and learning management, environmental and resource allocation, and engagement. The frequency and percentage for each dimension and level are reported in Table 4.

**Table 4:** Level of principal leadership and teacher self-efficacy.

Instrument	Dimension	Level (frequency/percentage)		
		Low	Middle	High
Principal leadership	Leadership mindset	13 (2.8%)	68 (14.8%)	378 (82.4%)
	Teaching and learning	14 (3.1%)	64 (13.9%)	381 (83.0%)
	Work environment	11 (2.4%)	56 (12.2%)	392 (85.4%)
	Teacher and student	10 (2.2%)	67 (14.6%)	382 (83.2%)
Teacher self-efficacy	Student engagement	4 (0.9%)	43 (9.4%)	412 (89.8%)
	Classroom management	4 (0.9%)	35 (7.6%)	420 (91.5%)

1.00–2.33, Low; 2.34–3.66, Middle; 3.67–5.00 High (Pallant, 2016).

### Correlation between job satisfaction and job stress

The Pearson product–moment correlation test was conducted to determine the relationship between principal leadership and teacher self-efficacy. As presented in Table 5, a significant positive correlation was observed between the two variables. Based on *Guilford’s Rule of Thumb* (Guilford, 1956), a correlation coefficient between 0.70 and 0.89 indicates a high correlation or strong relationship.

**Table 5:** Correlations between variables.

	Variables	1	2	3	4
1.	Leadership mindset				
2.	Teaching and learning	0.935**			
3.	Work environment	0.916**	0.948**		
4.	Teacher and student	0.876**	0.915**	0.950**	
5.	Teacher self-efficacy	0.754**	0.788**	0.837**	0.844**

**Correlation is significant at 0.01 (2-tailed).

### Influence of principal leadership on teacher self-efficacy

Regression analysis was performed to determine which dimensions of principal leadership (leadership vision, teaching and learning management, environmental and resource management, teacher engagement) significantly predicted teacher self-efficacy.

The analysis of variance (ANOVA) results presented in Table 6 indicated that the regression model was a good fit for the data, with a significance level of 0.000. The findings revealed a statistically significant relationship between principal leadership and teacher self-efficacy [*F*(4,454) = 303.52, *p* < 0.000]. Principal leadership explained approximately 72.8% of the variance in teacher self-efficacy (*R*^2^ = 0.728), while the remaining 27.2% was attributed to external factors that were not included in this study.

**Table 6:** Relationship between principal leadership and self-efficacy.

ANOVA	Sum of square	df	Mean square	*F*	Sig
Regression	146.51	4	36.628	303.52	0.000
Residual	54.78	454	0.121		
Total	201.30	458			

Model summary	*R*	*R* square	Adjusted *R* square	Std error of the estimate
Model 1	0.853	0.728	0.725	0.347

Coefficients	*B*	*β*	*t*	Sig *t*
Leadership mindset	0.048	0.058	0.815	0.416
Teaching and learning	0.073	0.094	1.03	0.303
Work environment	0.405	0.488	4.72	0.000
Teacher and student	0.420	0.518	6.51	0.000
Constant	1.404		14.72	0.000

Based on Table 6, the standardised beta values indicated that two dimensions of principal leadership significantly contributed to teacher self-efficacy: work environment (*β* = 0.488, *t* = 4.723; *p* = 0.000), and teacher and student engagement (*β* = 0.518, *t* = 6.509; *p* = 0.000). Specifically, a one-unit increase in the standard deviation of work environment resulted in a 0.488-unit increase in the standard deviation of teacher self-efficacy. Similarly, a one-unit increase in standard deviation of teacher and student engagement led to a 0.518-unit increase in the standard deviation of teacher self-efficacy. Overall, principal attitudes towards teacher and student engagement had the strongest influence on teacher self-efficacy.

## Discussion

The findings of this study provided a profound understanding of the relationship between principal leadership and teacher self-efficacy in the domains of student engagement and classroom management. Focusing on teachers in China, the study highlighted key dimensions of principal leadership that significantly influence teacher self-efficacy, providing valuable insights for improving school environments.

### High levels of teacher self-efficacy and principal leadership

Most respondents demonstrated high levels of self-efficacy, particularly in student engagement and classroom management. Similarly, the respondents rated principal leadership highly across all four dimensions: leadership mindset, teaching and learning, work environment, and teacher-student engagement. These findings are consistent with previous studies indicating that supportive and effective leadership strengthens teacher self-efficacy. For example, Tschannen-Moran and Hoy (2001) highlighted the significance of leader behaviours in enhancing teacher self-efficacy by providing supportive feedback and ensuring access to resources for effective teaching. Blase and Blase (1999) emphasised that principals who demonstrated instructional leadership qualities, such as promoting collaborations supporting professional development, significantly enhance teachers’ confidence in their instructional capabilities. Similarly, a systematic review by Leithwood et al. (2004) highlighted that effective school leadership enhances teachers’ commitment and strengthens their self-efficacy, leading to better student outcomes. Furthermore, the review highlighted that principals’ leadership practices are directly linked to teachers’ perceptions of their ability to promote student learning.

### Correlation between principal leadership and teacher self-efficacy

The results of the correlation analysis indicated a strong positive relationship between principal leadership and teacher self-efficacy. This highlighted the significant role that effective leadership plays in enhancing teachers’ confidence and performance. Notably, the dimensions of “Work Environment” and “Teacher and Student Engagement” emerged as the strongest correlations with teacher self-efficacy. These findings suggested that a supportive and resource-rich work environment, combined with a leadership style emphasising engagement and inclusivity, is essential in fostering higher levels of teacher self-efficacy. When principals cultivate an environment with adequate resources, collaborative practices, and meaningful engagement with both teachers and students, they empower teachers to feel valued and competent. Such an environment helps teachers navigate the complexities of modern educational challenges and motivates them to improve their instructional practices, ultimately enhancing overall student outcomes.

Studies consistently supported the assertion that principal leadership affects teacher self-efficacy, particularly through the dimensions identified in the present study. For example, Fackler and Malmberg (2016) indicated that a positive work environment significantly contributed to teachers’ sense of efficacy as it provides them with the necessary support and resources to perform effectively. Furthermore, Louis et al. (2010) emphasised that collaborative leadership practices that foster strong teacher-student relationships can enhance teachers’ self-efficacy and instructional effectiveness. Additionally, the work of Hallinger (2003) stressed on the crucial role of principal leadership in shaping the teachers’ organisational context. Hallinger’s findings indicated that supportive leadership practices, including fostering an open, engaging environment, are directly correlated with increased teacher confidence.

#### Impact of principal leadership on teacher self-efficacy

The findings underscore the critical role of principal leadership in shaping teacher self-efficacy, particularly through the dimensions of work environment and teacher-student engagement. These two leadership practices appear to foster an environment where teachers feel supported, empowered, and professionally respected. Drawing on Bandura’s (1997) self-efficacy theory, a well-structured work environment may serve simultaneously as social persuasion and vicarious experience, both of which are essential mechanisms in shaping individuals’ efficacy beliefs. When teachers operate in schools with clear instructional goals, collaborative cultures, and supportive leadership, they are more likely to believe in their ability to manage classrooms effectively and engage students meaningfully. Furthermore, the emphasis on teacher-student engagement reflects the cultural context of Chinese education, where harmonious relationships and moral authority are highly valued. This dimension may be particularly salient in Confucian-influenced educational systems, offering a culturally grounded explanation for its predictive strength. These findings build upon the work of Hallinger and Walker (2017) and Ma and Marion (2021) by demonstrating how leadership practices, when deeply embedded in specific cultural contexts, shape teachers’ self-efficacy beliefs within non-Western educational environments.

### Comparative analysis with existing studies

The results of the present study indicated a strong positive relationship between principal leadership and teacher self-efficacy, highlighting the critical role of supportive leadership in enhancing teacher performance. Among the dimensions assessed, “Work Environment” and “Teacher and Student Engagement” exhibited the strongest correlations with teacher self-efficacy. This underscores the importance of creating a resource-rich, inclusive work environment where teachers feel valued and supported. Such leadership practices enable teachers to perform effectively while addressing the challenges of modern teaching. These findings aligned with previous studies connecting supportive leadership to improved teacher outcomes. For example, Leithwood and Jantzi (2006) reported that principals who engage teachers in decision-making and promote a supportive work environment significantly enhance teacher efficacy and performance. Similarly, Louis et al. (2010) demonstrated that effective leadership practices, such as promoting collaboration and respectful working relationships, enhance teacher morale and effectiveness.

Furthermore, the study’s focus on “Teacher and Student Engagement” highlighted a culturally specific dimension of educational leadership. This aspect underscored the influence of Confucian values in Chinese education, which emphasised respect for hierarchical relationships and cultivation of harmonious interactions in professional settings. These cultural foundations implied that educational leadership in China must navigate the balance between traditional values and the adoption of modern practices that promote active participation and engagement from both teachers and students. Peng and Wang (2017) emphasised the critical role of Confucian values in shaping educational leadership practices in China. The study highlighted that respect for hierarchy and the prioritisation of relationships significantly influence the teacher-student and teacher-principal interactions, ultimately impacting teacher performance and self-efficacy.

### Unexplored variance and external factors

The finding that 27.2% of the variance in teacher self-efficacy remains unexplained indicated that while principal leadership is a significant factor, other variables also contribute to teachers’ self-efficacy. These may include personal attributes, such as individual characteristics and experiences, peer support systems, professional development opportunities, and the impact of policy interventions on teaching practices. These factors can significantly influence how teachers perceive effectiveness in classroom and student engagement, warranting further investigation. Research consistently emphasised that factors beyond leadership also impact teacher self-efficacy. For example, Tschannen-Moran and Hoy (2001) highlighted the role of personal characteristics and experiences in shaping self-efficacy beliefs. Their study indicated that teachers’ previous successes, emotional stability, and intrinsic motivation are factors that can enhance self-efficacy. Furthermore, Stone-Johnson (2016) highlighted the importance of peer support and collaboration in enhancing teacher confidence and reducing feelings of isolation. His research suggested that supportive peer relationships are key to enhancing teachers’ perceptions of their abilities and overall job satisfaction.

A comprehensive understanding of these additional variables is crucial for developing strategies that enhance teacher self-efficacy. Examining how personal attributes, such as intrinsic motivation and resilience, and external supports like peer collaboration and policy measures, contribute to teacher self-efficacy can help educational leaders and policymakers design more effective interventions for teacher development. These results align with Kunter et al. (2013), which demonstrated that structured professional development programmes and supportive school policies promote an environment that enhances teacher efficacy. The authors argued that systemic support, whether through leadership or policy frameworks, is critical to empowering teachers.

## Conclusion

In conclusion, the results of this study affirmed the critical influence of school leadership on teacher self-efficacy within the context of student engagement and classroom management. The high prevalence of self-efficacy among the teachers highlighted the crucial role of effective leadership practices in shaping teachers’ confidence and enhancing instructional effectiveness. The findings suggested that principal leadership, particularly in the dimensions of work environment and teacher-student relations, is essential for enhancing teacher efficacy and improving educational outcomes. Therefore, school leaders should focus on developing these aspects alongside fostering a positive school culture that supports both teachers and students.

The study enriched the existing literature by providing evidence from the Chinese educational context, emphasising the cultural and contextual nuances in leadership practices and teacher self-efficacy. Unlike previous studies which focused on general efficacy or Western frameworks (Cansoy and Parlar, 2018; Hallinger and Kovačević, 2019; Ma and Marion, 2021). This study introduces differentiated efficacy outcomes and links them to school leadership practices deeply shaped by Confucian values. As pointed out that leadership does not operate in isolation, rather, it is embedded within the organizational culture and structure (Ma and Marion, 2021). Furthermore, the findings of this study provided valuable insights for educational policymakers and school leaders aiming to elevate teaching quality and student success more effectively in East Asian schooling systems.

This study offers practical implications for educational leadership development and school improvement. First, given the significant impact of “working environment” and “teacher-student interaction” on teachers’ self-efficacy, principal training programmes should prioritise enhancing school leaders’ capacity to foster a supportive school climate. This includes strengthening principals’ communication skills, teacher empowerment strategies, and emotional intelligence to build trust and collaboration among staff. Second, as teacher-student interaction plays a crucial role, leadership development should also focus on equipping principals with the skills to promote relational teaching practices and cultivate a school culture that values meaningful teacher-student engagement. This can involve modelling respectful relationships, supporting students’ social–emotional learning, and encouraging open dialogue across the school community.

In addition, the findings underscore the importance of integrating culturally responsive content into principal preparation. In the Chinese educational context, leadership approaches aligned with Confucian relational values may be more effective in enhancing teachers’ confidence. Therefore, relevant modules should be incorporated to explore how cultural expectations shape leadership behaviours and influence teacher psychology. As noted above, by translating empirical insights into actionable strategies, leadership development initiatives can more effectively support school leaders in creating enabling work environments for teachers, ultimately contributing to improved instructional quality and student learning outcomes.

Future studies could employ longitudinal or mixed-method approaches to explore how principals’ instructional leadership impacts teacher efficacy over time. In addition, qualitative studies may offer deeper insights into the contextual and emotional factors underlying leadership-teacher dynamics, especially in diverse or rural settings. These approaches would enrich the current understanding of leadership’s influence on teaching practices and provide more nuanced evidence for designing targeted leadership interventions.

## Data Availability

The original contributions presented in the study are included in the article/supplementary material, further inquiries can be directed to the corresponding author.

## References

[ref1] Ahn J. Bowers A. J. (2024). Do teacher beliefs mediate leadership and teacher behaviors? Testing teacher self-efficacy’s mediation role between leadership for learning and teacher outcomes. J. Educ. Adm. 62, 197–222. doi: 10.1108/JEA-12-2022-0227

[ref2] Akram M. Kiran S. İlgan A. (2017). Development and validation of instructional leadership questionnaire. Int. J. Organ. Leadersh. 6, 73–88. doi: 10.33844/ijol.2017.60435

[ref3] Allinder R. M. (1994). The relationship between efficacy and the instructional practices of special education teachers and consultants. Teach. Educ. Spec. Educ. 17, 86–95.

[ref9001] Ashton P. T. Webb R. B. (1986). Making a difference: Teachers’ sense of efficacy and student achievement. New York: Longman.

[ref5] Balyer A. Özcan K. (2020). School principals’ instructional feedback to teachers: teachers’ views. Int. J. Curr. Instr. 12, 295–312.

[ref6] Bandura A. (1977). Self-efficacy: toward a unifying theory of behavioral change. Psychol. Rev. 84, 191–215. doi: 10.1037/0033-295X.84.2.191, PMID: 847061

[ref7] Bandura A. (1993). Perceived self-efficacy in cognitive development and functioning. Educ. Psychol. 28, 117–148. doi: 10.1207/s15326985ep2802_3

[ref9002] Bandura A. (1997). Self-efficacy: The exercise of control. New York: W.H. Freeman.

[ref9] Berkovich I. Hassan T. (2024). Principals’ digital instructional leadership during the pandemic: impact on teachers’ intrinsic motivation and students’ learning. Educ. Manag. Admin. Leadersh. 52, 934–954. doi: 10.1177/17411432221113411

[ref9003] Berman P. McLaughlin M. W. Bass-Golod G. V. Pauly E. Zellman G. L. (1977). Federal programs supporting educational change: Vol. VII: Factors affecting implementation and continuation. Santa Monica, CA: RAND Corporation.

[ref11] Blase J. Blase J. (1999). Principals’ instructional leadership and teacher’s self-efficacy. Educ. Adm. Q. 35, 329–349. doi: 10.1177/0013161X99353003

[ref12] Cansoy R. Parlar H. (2018). Examining the relationship between school principals’ instructional leadership behaviors, teacher self-efficacy, and collective teacher efficacy. Int. J. Educ. Manag. 32, 550–567. doi: 10.1108/IJEM-04-2017-0089

[ref13] Chen J. Lin C. Lin F. (2024). The interplay among EFL teachers’ emotional intelligence, self-efficacy, and burnout. Acta Psychol. 248:104364. doi: 10.1016/j.actpsy.2024.104364 38889657

[ref14] Daniëls E. Hondeghem A. Dochy F. (2019). A review on leadership and leadership development in educational settings. Educ. Res. Rev. 27, 110–125. doi: 10.1016/j.edurev.2019.02.003

[ref9004] Darling-Hammond L. Burns D. Campbell C. Goodwin A. L. Hammerness K. Low E. L.. (2017). Empowered educators: How high-performing systems shape teaching quality around the world. San Francisco, CA: Jossey-Bass (a Wiley brand).

[ref16] Donohoo J. (2018). Collective teacher efficacy research: productive patterns of behavior and other positive consequences. J. Educ. Change 19, 323–345. doi: 10.1007/s10833-018-9319-2

[ref17] Duan S. Bissaker K. Xu Z. (2024). Correlates of teachers’ classroom management self-efficacy: a systematic review and meta-analysis. Educ. Psychol. Rev. 36:43. doi: 10.1007/s10648-024-09881-2

[ref18] Duffin L. French B. Patrick H. (2012). The teachers’ sense of efficacy scale: confirming the factor structure with beginning pre-service teachers. Teach. Teach. Educ. 28, 827–834. doi: 10.1016/j.tate.2012.03.004

[ref19] Fackler S. Malmberg L. E. (2016). Teachers’ self-efficacy in 14 OECD countries: teacher, student group, school and leadership effects. Teach. Teach. Educ. 56, 185–195. doi: 10.1016/j.tate.2016.03.002

[ref20] Fimian M. J. Fastenau P. S. (1990). The validity and reliability of the teacher stress inventory: a re-analysis of aggregate data. J. Organ. Behav. 11, 151–157. doi: 10.1002/job.4030110206

[ref9005] Guilford J. P. (1956). Fundamental statistics in psychology and education (3rd ed.). New York, NY: McGraw-Hill.

[ref21] Guskey T. R. (1984). The influence of change in instructional effectiveness upon the affective characteristics of teachers. Am. Educ. Res. J. 21, 245–259. doi: 10.3102/00028312031003627

[ref22] Guskey T. R. Passaro P. D. (1994). Teacher efficacy: a study of construct dimensions. Am. Educ. Res. J. 31, 627–643.

[ref9006] Hallinger P. (2003). “Leading educational change: Reflections on the practice of instructional and transformational leadership” in International handbook of educational leadership and administration. eds. Walker A. Dimmock C., vol. 2 (Springer), 977–1000.

[ref24] Hallinger P. (2008). Methodologies for studying school leadership: a review of 25 years of research using the principal instructional management rating scale. In Annual meeting of the American Educational Research Association, New York (48).

[ref25] Hallinger P. (2011). Leadership for learning: lessons from 40 years of empirical research. J. Educ. Adm. 49, 125–142. doi: 10.1108/09578231111116699

[ref26] Hallinger P. Heck R. H. (1996). “The principal’s role in school effectiveness: an assessment of methodological progress, 1980–1995” in International handbook of educational leadership and administration: part 1–2 (Dordrecht: Springer Netherlands), 723–783.

[ref27] Hallinger P. Heck R. H. (2010). Collaborative leadership and school improvement: understanding the impact on school capacity and student learning. School Leadersh. Manag. 30, 95–110. doi: 10.1080/13632431003663214

[ref28] Hallinger P. Kovačević J. (2019). A bibliometric review of research on educational administration: science mapping the literature, 1960 to 2018. Rev. Educ. Res. 89, 335–369. doi: 10.3102/0034654319830380

[ref29] Hallinger P. Walker A. (2017). Leading learning in Asia–emerging empirical insights from five societies. J. Educ. Adm. 55, 130–146. doi: 10.1108/JEA-02-2017-0015

[ref30] Heck R. H. Larsen T. J. Marcoulides G. A. (1990). Instructional leadership and school achievement: validation of a causal model. Educ. Adm. Q. 26, 94–125. doi: 10.1177/0013161X90026002002

[ref31] Hettinger K. Lazarides R. Schiefele U. (2024). Longitudinal relations between teacher self-efficacy and student motivation through matching characteristics of perceived teaching practice. Eur. J. Psychol. Educ. 39, 1299–1325. doi: 10.1007/s10212-023-00744-y

[ref32] Husain A. N. Miller L. C. Player D. W. (2021). Principal turnover: using teacher-assessments of principal quality to understand who leaves the principalship. Educ. Adm. Q. 57, 683–715. doi: 10.1177/0013161X211011235

[ref9007] Kasalak G. Dagyar M. (2020). The relationship between teacher self-efficacy and teacher job satisfaction: A meta-analysis of the teaching and learning international survey (TALIS). Educ. Sci.: Theory and Prac 20, 16–33. doi: 10.12738/jestp.2020.3.002

[ref9008] Kiernan D. (2014). Natural resources biometrics. Open SUNY Textbooks, Milne Lib rary, State University of New York at Geneseo. Geneseo, NY, USA.

[ref35] Klassen R. M. Chiu M. M. (2011). The occupational commitment and intention to quit of practicing and pre-service teachers: influence of self-efficacy, job stress, and teaching context. Contemp. Educ. Psychol. 36, 114–129. doi: 10.1016/j.cedpsych.2011.01.002

[ref36] Klassen R. M. Tze V. M. (2014). Teachers’ self-efficacy, personality, and teaching effectiveness: a meta-analysis. Educ. Res. Rev. 12, 59–76. doi: 10.1016/j.edurev.2014.06.001

[ref37] Kunter M. Klusmann U. Baumert J. Richter D. Voss T. Hachfeld A. (2013). Professional competence of teachers: effects on instructional quality and student development. J. Educ. Psychol. 105, 805–820. doi: 10.1037/a0032583

[ref38] Lazarides R. Watt H. M. Richardson P. W. (2020). Teachers’ classroom management self-efficacy, perceived classroom management and teaching contexts from beginning until mid-career. Learn. Instr. 69:101346. doi: 10.1016/j.learninstruc.2020.101346

[ref9009] Leithwood K. Harris A. Hopkins D. (2008). Seven strong claims about successful school leadership. School leadership and management 28, 27–42.

[ref9010] Leithwood K. Seashore Louis K. Anderson S. Wahlstrom K. (2004). Review of research: How leadership influences student learning. New York: The Wallace Foundation. Avaliable online at: https://www.wallacefoundation.org/knowledge-center/Documents/How-Leadership-Influences-Student-Learning.pdf

[ref40] Leithwood K. Jantzi D. (2005). A review of transformational school leadership research 1996–2005. Leadersh. Policy Sch. 4, 177–199. doi: 10.1080/15700760500244769

[ref41] Leithwood K. Jantzi D. (2006). Transformational school leadership for large-scale reform: effects on students, teachers, and their classroom practices. Sch. Eff. Sch. Improv. 17, 201–227. doi: 10.1080/09243450600565829

[ref42] Leithwood K. Sun J. (2018). Academic culture: a promising mediator of school leaders’ influence on student learning. J. Educ. Adm. 56, 350–363. doi: 10.1108/JEA-01-2017-0009

[ref62] Liu S. N. Hallinger P. (2021). Unpacking the effects of culture on school leadership and teacher learning in China. Educ. Manag. Adm. Leadersh. 49, 214–233. doi: 10.1177/1741143219896042

[ref44] Liu Y. Li L. Huang C. (2022). To what extent is shared instructional leadership related to teacher self-efficacy and student academic performance in China? Sch. Eff. Sch. Improv. 33, 381–402. doi: 10.1080/09243453.2022.2029746

[ref9012] Louis K. S. Leithwood K. Wahlstrom K. L. Anderson S. (2010). Investigating the links to improved student learning: Final report of research findings. New York, NY: The Wallace Foundation.

[ref46] Ma X. Marion R. (2021). Exploring how instructional leadership affects teacher efficacy: a multilevel analysis. Educ. Manag. Admin. Leadersh. 49, 188–207. doi: 10.1177/1741143219888742

[ref47] Mertkan S. Arsan N. Inal Cavlan G. Onurkan Aliusta G. (2017). Diversity and equality in academic publishing: the case of educational leadership. Compare J. Comp. Int. Educ. 47, 46–61. doi: 10.1080/03057925.2015.1136924

[ref48] Mok M. M. C. Moore P. J. (2019). Teachers & self-efficacy. Educ. Psychol. 39, 1–3. doi: 10.1080/01443410.2019.1567070

[ref49] Morris J. E. Lummis G. W. Lock G. Ferguson C. Hill S. Nykiel A. (2020). The role of leadership in establishing a positive staff culture in a secondary school. Educ. Manag. Admin. Leadersh. 48, 802–820. doi: 10.1177/1741143219864937

[ref9013] Moss C. M. Brookhart S. M. (2019). Advancing formative assessment in every classroom: A guide for instructional leaders. Alexandria, VA: ASCD.

[ref51] Murphy J. (1988). The instructional leadership role of the school principal: an analysis. Educ. Eval. Policy Anal. 10, 71–79.

[ref52] Murphy J. F. Goldring E. B. Cravens X. C. Elliott S. N. Porter A. C. (2007). The Vanderbilt assessment of leadership in education: measuring learning-centered leadership. J. East China Normal Univ. 29, 1–10.

[ref9014] Pallant J. (2016). SPSS survival manual: A step by step guide to data analysis using IBM SPSS (6th ed.). Berkshire, England: McGraw-Hill Education.

[ref54] Peng X. Q. Wang T. (2017). Investigating Chinese educational leaders’ Confucian ethics and value orientations in a transnational leadership program. Contemp. Educ. Res. Q. 25, 45–78.

[ref55] Redondo-Sama G. Khaqan S. Morlà-Folch T. Munté-Pascual A. (2025). Leading schools through dialogue: the role of principals in schools as learning communities. J. New Approach. Educ. Res. 14:12. doi: 10.1007/s44322-025-00033-0

[ref56] Röhl S. Pietsch M. Cramer C. (2024). School leaders’ self-efficacy and its impact on innovation: findings of a repeated measurement study. Educ. Manag. Admin. Leadersh. 52, 1477–1496. doi: 10.1177/17411432221132482

[ref57] Sebastian J. Allensworth E. (2012). The influence of principal leadership on classroom instruction and student learning: a study of mediated pathways to learning. Educ. Adm. Q. 48, 626–663. doi: 10.1177/0013161X11436273

[ref58] Sellami A. Santhosh M. E. Michaleczek I. Alazaizeh M. Madad J. (2025). Unveiling teachers’ instructional self-efficacy in science, mathematics, and technology: personal and contextual influences. Can. J. Sci. Math. Technol. Educ. 24, 418–438. doi: 10.1007/s42330-025-00359-z

[ref59] Sergiovanni T. J. (1990). Adding value to leadership gets extraordinary results. Educ. Leadersh. 47, 23–27.

[ref9015] Sergiovanni T. J. (1984). Leadership and Excellence in Schooling. Educational Leadership 41, 4–6.

[ref9016] Shah D. (2023). Teachers’ self-efficacy and classroom management practices: A theoretical study. Journal of Education and Research 13, 8–26. doi: 10.51474/jer.v13i1.661

[ref9017] Soodak L. C. Podell D. M. (1993). Teacher efficacy and student problem as factors in special education referral. The Journal of Special Education 27, 66–81.

[ref61] Stone-Johnson C. (2016). Intensification and isolation: alienated teaching and collaborative professional relationships in the accountability context. J. Educ. Chang. 17, 29–49. doi: 10.1007/s10833-015-9255-3

[ref63] Siaw Y.-L. Zhang L. Jiang N. (2021). Development and validation of the instructional leadership scale for Chinese primary and secondary school principals. Educ. Observ. 10, 12–17.

[ref64] Spillane J. P. Diamond J. B. Walker L. J. Halverson R. Jita L. (2001). Urban school leadership for elementary science instruction: identifying and activating resources in an undervalued school subject. J. Res. Sci. Teach. 38, 918–940. doi: 10.1002/tea.1039

[ref65] Supovitz J. Sirinides P. May H. (2010). How principals and peers influence teaching and learning. Educ. Adm. Q. 46, 31–56. doi: 10.1177/1094670509353043

[ref66] Tschannen-Moran M. Gareis C. R. (2015). Principals, trust, and cultivating vibrant schools. Societies 5, 256–276. doi: 10.3390/soc5020256

[ref67] Tschannen-Moran M. Hoy A. W. Hoy W. K. (1998). Teacher efficacy: its meaning and measure. Rev. Educ. Res. 68, 202–248. doi: 10.3102/00346543068002202

[ref68] Tschannen-Moran M. Hoy A. W. (2001). Teacher efficacy: capturing an elusive construct. Teach. Teach. Educ. 17, 783–805. doi: 10.1016/S0742-051X(01)00036-1

[ref69] Wang M. T. Degol J. L. (2016). School climate: a review of the construct, measurement, and impact on student outcomes. Educ. Psychol. Rev. 28, 315–352. doi: 10.1007/s10648-015-9319-1

[ref9018] Wang Y. Pan Z. (2023). Modeling the effect of Chinese EFL teachers’ self-efficacy and resilience on their work engagement: A structural equation modeling analysis. Sage Open. 13:21582440231214329.

[ref71] Wayman J. C. Stringfield S. (2006). Data use for school improvement: school practices and research perspectives. Am. J. Educ. 112, 463–468. doi: 10.1086/505055

[ref9019] Witte R. S. Witte J. S. (2017). Statistics (11th ed.). Hoboken, NJ: Wiley.

[ref74] Wong L. N. (2003). Changing roles and shifting authority of principals in China: a mixed role of manager and clan leader. Educ. Soc. 21, 37–54. doi: 10.7459/es/21.2.04

[ref75] Zee M. Koomen H. M. Y. (2016). Teacher self-efficacy and its effects on classroom processes. Rev. Educ. Res. 86, 981–1015. doi: 10.3102/0034654315626801

